# Effect of biofeedback electrical stimulation combined with HoLEP on surgical outcomes in patients with benign prostatic hyperplasia complicated with detrusor underactivity: a retrospective cohort study

**DOI:** 10.3389/fsurg.2026.1847062

**Published:** 2026-05-25

**Authors:** Tengfei Gu, Jie Li, Ting Chen, Yongtao Pan, Qinzhou Yu, Jing Sha

**Affiliations:** 1 Department of Urology, Lishui Municiple Central Hospital, The Fifth Afliated Hospital of Wenzhou Medical University, Lishui, China; 2 Department of Nursing, Lishui Municiple Central Hospital, The Fifth Afliated Hospital of Wenzhou Medical University, Lishui, China

**Keywords:** benign prostatic hyperplasia, biofeedback electrical stimulation, detrusor underactivity, holmium laser enucleation of the prostate, voiding function

## Abstract

**Objective:**

To investigate the clinical efficacy and safety of biofeedback electrical stimulation combined with holmium laser enucleation of the prostate (HoLEP) in the treatment of patients with benign prostatic hyperplasia (BPH) complicated by detrusor underactivity (DUA).

**Methods:**

A retrospective analysis was conducted on 100 patients with BPH and DUA who had surgical indications and were treated in the Department of Urology of our hospital from January 2023 to June 2025. Patients were divided into an intervention group (*n* = 51) and a control group (*n* = 49) according to the treatment modality they received. Patients in the intervention group underwent HoLEP followed by biofeedback electrical stimulation therapy (three times per week for a total of 10 sessions), whereas those in the control group received HoLEP alone. The International Prostate Symptom Score (IPSS), Quality of Life score (QOL), maximum urinary flow rate (Qmax), bladder contractility index (BCI), bladder outlet obstruction index (BOOI), maximum detrusor pressure (Pdetmax), post-void residual volume (PVR), voiding efficiency (VE), and postoperative complications were compared between the two groups before surgery and at 3 months postoperatively.

**Results:**

Baseline characteristics were comparable between the two groups (*P* > 0.05). At 3 months postoperatively, the intervention group showed significantly higher Qmax (14.38 ± 1.47 mL/s vs. 10.01 ± 0.85 mL/s, *P* < 0.001) and BCI (111.68 ± 10.15 vs. 93.96 ± 8.42, *P* < 0.001), significantly lower IPSS (10.8 ± 1.9 vs. 18.6 ± 2.1, *P* < 0.001) and QOL scores (2.1 ± 0.8 vs. 3.0 ± 0.6, *P* < 0.001), significantly lower PVR (21.8 ± 5.8 mL vs. 40.2 ± 7.5 mL, *P* < 0.001), and significantly higher VE (77.8 ± 6.2% vs. 61.9 ± 5.8%, *P* < 0.001) compared with the control group. The proportion of patients achieving Qmax ≥15 mL/s at 3 months postoperatively was 39.2% in the intervention group vs. 20.8% in the control group (*P* = 0.022). At 90 days postoperatively, the incidence rates of urinary tract infection (13.7% vs. 28.6%, *P* = 0.047), urinary incontinence (9.8% vs. 24.5%, *P* = 0.039), and indwelling catheter reinsertion (2.0% vs. 12.2%, *P* = 0.037) were significantly lower in the intervention group than in the control group. No significant differences were observed in the incidence of postoperative bleeding or urethral stricture between the two groups (*P* > 0.05).

**Conclusion:**

Biofeedback electrical stimulation combined with HoLEP significantly improves voiding function, clinical symptoms, and quality of life in patients with BPH and DUA, enhances bladder contractility, and reduces the risk of postoperative complications, offering clear clinical benefits and a favorable safety profile, warranting broader clinical adoption.

## Introduction

Benign prostatic hyperplasia (BPH) is the most common urological condition in elderly men, affecting approximately 30% of men over 50 years of age and up to 90% of those aged 80 years and older (1). Detrusor underactivity (DUA) represents another major contributor to lower urinary tract symptoms, with a reported prevalence of 37%–47% among men with BPH (2, 3). In this patient population, simply relieving obstruction through holmium laser enucleation of the prostate (HoLEP) may still leave residual issues such as poor voiding efficiency and increased post-void residual volume due to inadequate detrusor contractility, thereby compromising surgical outcomes (4).

To date, no universally effective treatment for DUA has been established. Biofeedback electrical stimulation (BFES) has been shown to effectively improve pelvic floor muscle function and has demonstrated favorable results in patients with pelvic floor dysfunction and post-prostatectomy urinary incontinence (5). However, whether BFES can improve surgical outcomes in patients with BPH and DUA remains supported by limited high-quality evidence. The present study retrospectively analyzed patients with BPH and DUA to evaluate the clinical efficacy and safety of HoLEP combined with BFES, aiming to provide a novel treatment strategy for this challenging patient population.

## Patients and methods

### Study design and patient population

This retrospective cohort study was approved by the institutional ethics committee. A total of 120 patients with surgical indications for BPH and DUA who presented to the Department of Urology of our hospital between January 2023 and June 2025 were initially enrolled. Based on the treatment modality received, patients were allocated to either the intervention group (BFES combined with HoLEP) or the control group (HoLEP alone), with 60 patients in each group. Inclusion criteria were: age ≥55 years, urodynamic diagnosis of bladder outlet obstruction (BOOI ≥20) with concomitant DUA (BCI <100), and fulfillment of surgical indications for BPH. Exclusion criteria included a history of severe pelvic floor dysfunction, neurological disorders, urethral stricture or prior urethral surgery, postoperative pathological diagnosis of prostate cancer, and incomplete clinical data.

### Surgical procedure and intervention

All surgeries were performed by the same team of senior surgeons using a Holmium laser system (Lumenis, Israel) with the following parameters: power 80 W, frequency 40 Hz, and energy 2.0 J. The enucleation was carried out using the three-lobe technique. Postoperatively, a 20-Fr Foley catheter was placed, and continuous bladder irrigation was maintained until the effluent was clear, after which the catheter was removed.

Patients in the intervention group received BFES postoperatively using a biofeedback electrical stimulator (Marlund Medical, China). Electrodes were positioned over the suprapubic bladder region and the inguinal area. The stimulation parameters were as follows: frequency 50 Hz, pulse duration 300 μs, and on/off ratio 1:1. Stimulation intensity was gradually increased from 0 mA to the maximum tolerated intensity (not exceeding 50 mA). Each session lasted 30 min and was performed three times per week for a total of 10 sessions. During treatment, biofeedback training was provided simultaneously to guide pelvic floor muscle contraction. Patients in the control group underwent HoLEP alone without postoperative BFES.

### Study variables

The following variables were collected and analyzed:

- Baseline characteristics: Age, height, weight, body mass index (BMI), American Society of Anesthesiologists (ASA) classification, presence of hypertension, diabetes mellitus, coronary artery disease, cerebral infarction, serum prostate-specific antigen (PSA) level, prostate volume, intravesical prostatic protrusion, presence of bladder stones, preoperative pelvic floor muscle score, preoperative IPSS, preoperative QOL score, and preoperative urinary incontinence status.
- Urodynamic parameters: Bladder contractility index (BCI), bladder outlet obstruction index (BOOI), maximum urinary flow rate (Qmax), maximum detrusor pressure (Pdetmax), free Qmax, post-void residual volume (PVR), and voiding efficiency (VE) were assessed preoperatively and at 3 months postoperatively using a urodynamic system.
- Symptom scores and quality of life: IPSS and QOL scores were recorded.
- Perioperative parameters: Operative time, intraoperative irrigation volume, hemoglobin decrease, postoperative white blood cell count, postoperative C-reactive protein level, and mean pain score on postoperative day 3 (visual analog scale, VAS).
- Postoperative complications within 90 days: Urinary tract infection, urinary incontinence, indwelling catheter reinsertion, bleeding, and urethral stricture.

### Statistical analysis

Statistical analyses were performed using SPSS version 22.0 (IBM Corp., Armonk, NY, USA). Normally distributed continuous variables were expressed as mean ± standard deviation (SD) and compared using the independent-samples t-test. Non-normally distributed continuous variables were expressed as median (minimum–maximum) and analyzed using the Mann–Whitney *U* test. Categorical variables were presented as frequencies (percentages) and compared using the chi-square test or Fisher's exact test, as appropriate. The intragroup comparisons of preoperative and postoperative parameters were performed using the paired *t*-test. A two-tailed *P*-value <0.05 was considered statistically significant.

## Results

**Figure 1:**
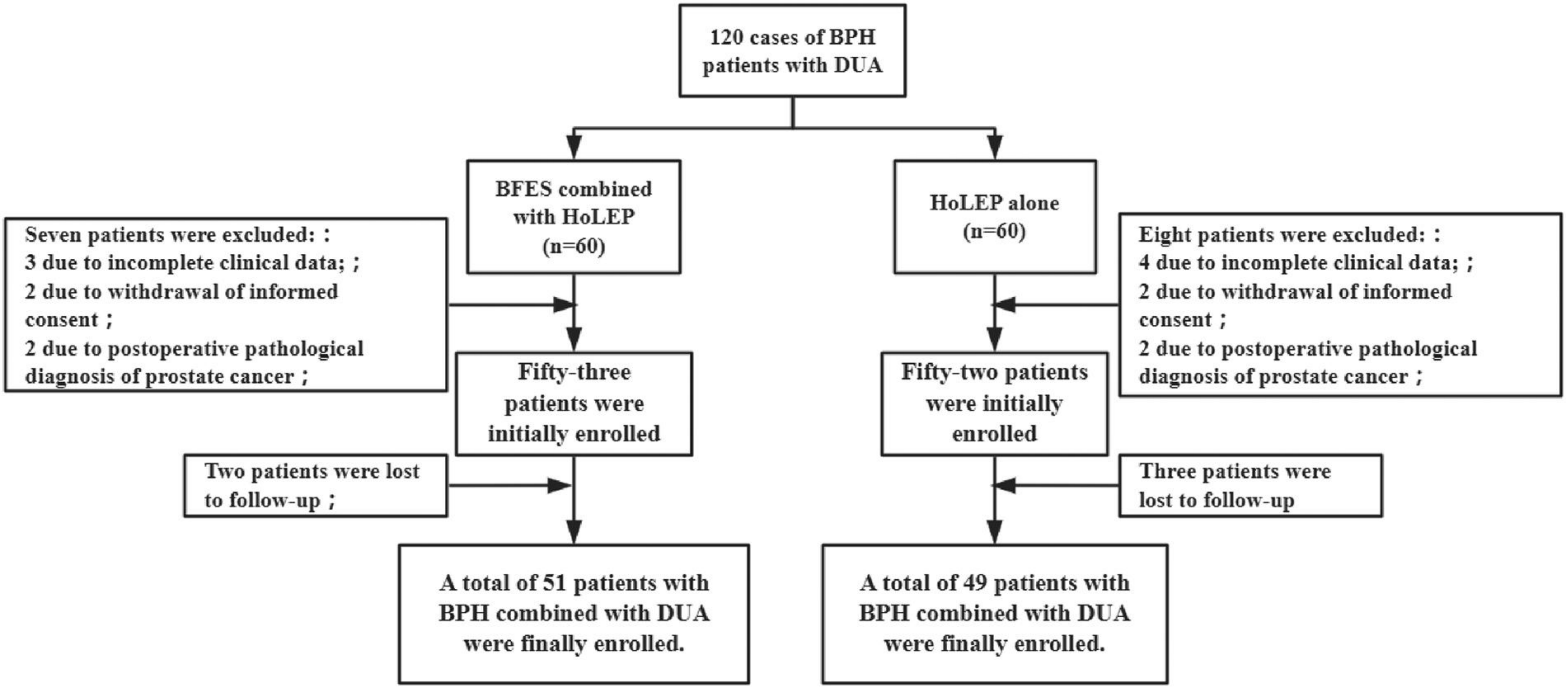
Flowchart of patient enrollment and exclusion in this study.

Following application of the exclusion criteria, 7 patients were excluded from the intervention group (3 due to incomplete clinical data, 2 due to withdrawal of informed consent, and 2 due to postoperative pathological diagnosis of prostate cancer), and 8 patients were excluded from the control group (4 due to incomplete clinical data, 2 due to withdrawal of informed consent, and 2 due to postoperative pathological diagnosis of prostate cancer). At the 3-month postoperative follow-up, an additional 2 patients from the intervention group and 3 patients from the control group were excluded because they did not complete the follow-up. The final analysis included 51 patients in the intervention group (BFES combined with HoLEP) and 49 patients in the control group (HoLEP alone). A flowchart of patient enrollment and exclusion is presented in Figure 1.

### Baseline characteristics

No statistically significant differences were observed between the two groups in terms of age, height, weight, BMI, ASA classification, comorbidities (hypertension, diabetes mellitus, coronary artery disease, cerebral infarction), serum PSA level, prostate volume, intravesical prostatic protrusion, presence of bladder stones, preoperative pelvic floor muscle score, preoperative IPSS, preoperative QOL score, preoperative urinary incontinence status, or urodynamic parameters (BCI, BOOI, Qmax, Pdetmax, PVR, VE) (*P* > 0.05 for all) (Table 1).

**Table 1:** Comparison of baseline characteristics between the two groups [mean ± SD, *n* (%)].

Variable	Intervention Group (*n* = 51)	Control Group (*n* = 49)	*P* value
Age (years)	69.25 ± 2.78	69.31 ± 2.69	0.91
Height (cm)	164.90 ± 4.10	165.60 ± 4.30	0.31
Weight (kg)	69.50 ± 6.00	71.20 ± 5.80	0.21
BMI (kg/m²)	25.48 ± 2.30	25.92 ± 2.20	0.33
ASA grade I–II, *n* (%)	34 (66.7)	31 (63.3)	0.73
Hypertension, *n* (%)	27 (52.9)	27 (55.1)	0.83
Diabetes mellitus, *n* (%)	12 (23.5)	12 (24.5)	0.91
Coronary artery disease, *n* (%)	11 (21.6)	11 (22.4)	0.92
Cerebral infarction, *n* (%)	9 (17.6)	10 (20.4)	0.73
tPSA (ng/mL)	3.82 ± 0.98	4.18 ± 1.05	0.13
Prostate volume (mL)	62.80 ± 12.10	65.40 ± 11.80	0.36
Intravesical prostatic protrusion (mm)	10.60 ± 3.80	11.30 ± 3.60	0.40
Bladder stones, *n* (%)	12 (23.5)	13 (26.5)	0.73
Preoperative pelvic floor muscle score	72.40 ± 3.10	72.30 ± 3.20	0.83
Preoperative IPSS	25.30 ± 2.80	25.00 ± 2.70	0.56
Preoperative QOL score	4.50 ± 1.10	4.40 ± 1.00	0.47
Preoperative urinary incontinence, *n* (%)	10 (19.6)	11 (22.4)	0.73
Preoperative BCI	81.20 ± 7.80	79.40 ± 7.50	0.21
Preoperative BOOI	52.40 ± 7.80	50.90 ± 7.50	0.28
Preoperative Qmax (mL/s)	6.80 ± 1.10	6.70 ± 1.00	0.56
Preoperative Pdetmax (cmH_2_O)	46.80 ± 6.20	46.30 ± 6.40	0.73
Preoperative PVR (mL)	125.60 ± 28.40	122.80 ± 26.90	0.54
Preoperative VE (%)	48.20 ± 6.20	47.80 ± 6.50	0.71

BCI, bladder contractility index; BOOI, bladder outlet obstruction index; Qmax, maximum urinary flow rate; Pdetmax, maximum detrusor pressure; PVR, post-void residual volume; VE, voiding efficiency; PSA, prostate-specific antigen; ASA, American Society of Anesthesiologists; IPSS, International Prostate Symptom Score; QOL, quality of life.

### Perioperative data

Comparison of perioperative parameters revealed that operative time, intraoperative irrigation volume, hemoglobin decrease, postoperative white blood cell count, postoperative C-reactive protein level, mean pain score at postoperative day 3, and postoperative hospital stay were slightly higher in the intervention group than in the control group, but none of these differences reached statistical significance (*P* > 0.05 for all) (Table 2).

**Table 2:** Comparison of perioperative data between the two groups (mean ± SD).

Variable	Intervention Group (*n* = 51)	Control Group (*n* = 49)	*P* value
Operative time (min)	60.35 ± 8.92	57.97 ± 6.88	0.15
Intraoperative irrigation volume (L)	17.12 ± 4.56	16.28 ± 3.17	0.30
Preoperative hemoglobin (g/L)	121.05 ± 11.23	119.98 ± 11.34	0.64
Postoperative hemoglobin (g/L)	108.46 ± 9.87	109.84 ± 9.37	0.48
Hemoglobin decrease (g/L)	13.58 ± 6.35	12.34 ± 4.94	0.28
Postoperative WBC (×10⁹/L)	10.65 ± 2.03	10.02 ± 1.51	0.09
Duration of postoperative irrigation (days)	1.58 ± 0.61	1.55 ± 0.50	0.79
Postoperative irrigation volume (L)	13.58 ± 3.45	13.09 ± 3.06	0.46
Time to catheter removal (days)	2.52 ± 0.58	2.48 ± 0.50	0.72
Postoperative hospital stay (days)	3.62 ± 0.78	3.56 ± 0.59	0.67
Mean VAS score at postoperative day 3	2.35 ± 0.52	2.24 ± 0.41	0.24

WBC, white blood cell; VAS, visual analog scale.

### Urodynamic parameters

No significant differences were observed in preoperative urodynamic parameters (Qmax, BCI, BOOI, Pdetmax, PVR, VE) between the two groups (*P* > 0.05 for all). At 3 months postoperatively, the intervention group demonstrated significantly higher Qmax (14.38 ± 1.47 mL/s vs. 10.01 ± 0.85 mL/s, *P* < 0.001), BCI (111.68 ± 10.15 vs. 93.96 ± 8.42, *P* < 0.001), and VE (77.8 ± 6.2% vs. 61.9 ± 5.8%, *P* < 0.001), as well as significantly lower BOOI (17.8 ± 3.2 vs. 23.2 ± 2.5, *P* < 0.001), Pdetmax (40.2 ± 4.1 cmH_2_O vs. 44.5 ± 4.2 cmH_2_O, *P* < 0.001), and PVR (21.8 ± 5.8 mL vs. 40.2 ± 7.5 mL, *P* < 0.001) compared with the control group (Table 3).

**Table 3:** Comparison of urodynamic parameters between the two groups (mean ± SD).

Parameter	Time Point	Intervention Group (*n* = 51)	Control Group (*n* = 49)	Intra-group *P* value ①	Inter-group *P* value ②
Qmax (mL/s)	Preoperative	6.80 ± 1.10	6.70 ± 1.00		0.56
	Postoperative 3 mo	14.38 ± 1.47	10.01 ± 0.85	<0.001/<0.001	<0.001
Qmax > 15 mL/s [*n* (%)]	Postoperative 3 mo	20 (39.2)	10 (20.8)		0.022
BCI	Preoperative	81.20 ± 7.80	79.40 ± 7.50		0.21
	Postoperative 3 mo	111.68 ± 10.15	93.96 ± 8.42	<0.001/<0.001	<0.001
BOOI	Preoperative	52.40 ± 7.80	50.90 ± 7.50		0.28
	Postoperative 3 mo	17.80 ± 3.20	23.20 ± 2.50	<0.001/<0.001	<0.001
Pdetmax (cmH₂O)	Preoperative	46.80 ± 6.20	46.30 ± 6.40		0.73
	Postoperative 3 mo	40.20 ± 4.10	44.50 ± 4.20	<0.001/0.025	<0.001
PVR (mL)	Preoperative	125.60 ± 28.40	122.80 ± 26.90		0.54
	Postoperative 3 mo	21.80 ± 5.80	40.20 ± 7.50	<0.001/<0.001	<0.001
VE (%)	Preoperative	48.20 ± 6.20	47.80 ± 6.50		0.71
	Postoperative 3 mo	77.80 ± 6.20	61.90 ± 5.80	<0.001/<0.001	

① Intra-group *P* values represent comparisons between preoperative and 3-month postoperative values within the intervention and control groups, respectively. ② Inter-group *P* values represent comparisons between the two groups. Qmax, maximum urinary flow rate; BCI, bladder contractility index; BOOI, bladder outlet obstruction index; Pdetmax, maximum detrusor pressure; PVR, post-void residual volume; VE, voiding efficiency.

### Pelvic floor muscle score, IPSS, and QOL score

No significant differences were observed in preoperative pelvic floor muscle score, IPSS, or QOL score between the two groups (*P* > 0.05). At 3 months postoperatively, the intervention group had a significantly higher pelvic floor muscle score (*P* < 0.001) and significantly lower IPSS and QOL scores (*P* < 0.001 for both) compared with the control group (Table 4).

**Table 4:** Comparison of pelvic floor muscle score, IPSS, and QOL score between the two groups (mean ± SD).

Parameter	Time Point	Intervention Group (*n* = 51)	Control Group (*n* = 49)	Intra-group *P* value ①	Inter-group *P* value ②
Pelvic floor muscle score	Preoperative	72.40 ± 3.10	72.30 ± 3.20		0.83
	Postoperative 3 mo	84.20 ± 2.90	73.50 ± 3.40	<0.001/0.06	<0.001
IPSS	Preoperative	25.30 ± 2.80	25.00 ± 2.70		0.56
	Postoperative 3 mo	7.30 ± 2.40	10.60 ± 3.70	<0.001/<0.001	<0.001
QOL score	Preoperative	4.50 ± 1.10	4.40 ± 1.00		0.47
	Postoperative 3 mo	1.60 ± 0.70	2.50 ± 1.10	<0.001/<0.001	<0.001

① Intra-group *P* values represent comparisons between preoperative and 3-month postoperative values within the intervention and control groups, respectively. ② Inter-group *P* values represent comparisons between the two groups. Pelvic floor muscle score was assessed using the Marlund Pelvic Floor Muscle Evaluation System (full score: 100). IPSS, International Prostate Symptom Score (range: 0–35); QOL, quality of life score (range: 0–6).

### Postoperative adverse events

The intervention group had significantly lower incidences of postoperative urinary tract infection, urinary incontinence, and indwelling catheter reinsertion compared with the control group (*P* < 0.05 for all). No significant differences were observed in the rates of bleeding or urethral stricture between the two groups (*P* > 0.05) (Table 5).

**Table 5:** Comparison of postoperative adverse events between the two groups [*n* (%)].

Adverse event	Intervention Group (*n* = 51)	Control Group (*n* = 49)	*P* value
Urinary tract infection	7 (13.7)	14 (28.6)	0.047
Urinary incontinence	5 (9.8)	12 (24.5)	0.039
Indwelling catheter reinsertion	1 (2.0)	6 (12.2)	0.037
Bleeding	1 (2.0)	2 (4.1)	0.54
Urethral stricture	0 (0)	2 (4.1)	0.14

### Multivariate logistic regression analysis

After adjusting for age, preoperative Qmax, and prostate volume, treatment modality remained an independent predictor of achieving Qmax >15 mL/s at 3 months postoperatively (OR = 3.27, 95% CI: 1.26–8.49, *P* = 0.014) (Table 6).

**Table 6:** Multivariate logistic regression analysis (dependent variable: Qmax >15 mL/s at 3 months postoperatively).

Variable	*β*	SE	Wald *χ*^2^	OR (95% CI)	*P* value
Treatment modality (intervention vs. control)	1.186	0.485	5.98	3.27 (1.26–8.49)	0.014
Age (per 1-year increase)	−0.042	0.058	0.52	0.96 (0.85–1.08)	0.47
Preoperative Qmax (per 1 mL/s increase)	0.312	0.189	2.72	1.37 (0.94–1.99)	0.10
Prostate volume (per 1 mL increase)	−0.018	0.016	1.27	0.98 (0.95–1.01)	0.26
Constant	−2.153	2.081	1.07	0.12	0.30

Hosmer–Lemeshow test: *χ*^2^ = 5.23, *P* = 0.73, indicating good model fit.

## Discussion

In this retrospective analysis of 100 patients with BPH and DUA, we systematically compared the efficacy of postoperative BFES combined with HoLEP vs. HoLEP alone. The results demonstrated that the combination therapy group achieved significantly better outcomes at 3 months postoperatively in terms of voiding function parameters (Qmax, BCI, VE), symptom scores (IPSS, QOL), and complication rates (urinary tract infection, urinary incontinence, recurrent urinary retention), without increasing the perioperative risk of bleeding. These findings provide a practical and promising therapeutic strategy for the challenging clinical scenario of BPH with concomitant DUA.

In the present study, the postoperative BCI in the intervention group (111.68 ± 10.15) was significantly higher than that in the control group (93.96 ± 8.42) and exceeded the diagnostic threshold for DUA (100), whereas the control group remained within the DUA range. This difference carries important pathophysiological implications. The BCI, calculated as PdetQmax + 5Qmax, integrates both voiding pressure and flow rate, serving as a key indicator of detrusor contractility (6). While surgery alone relieves the mechanical obstruction at the bladder outlet, it does not reverse the detrusor dysfunction that develops secondary to chronic obstruction. BFES, in contrast, may exert its effects through two mechanisms: first, electrical stimulation directly activates the detrusor muscle, facilitating the recovery of its contractile function; second, biofeedback training helps patients perceive and control their pelvic floor muscles, thereby improving neuromuscular coordination during voiding (7, 8). A randomized controlled trial by Ladi-Seyedian et al. (9) in children with non-neurogenic DUA similarly demonstrated that biofeedback therapy significantly improved BCI and voiding efficiency. The present study extends this mechanistic insight to an elderly post-BPH surgery population, further validating the role of BFES in enhancing detrusor function.

Notably, the postoperative Qmax in the intervention group reached 14.38 ± 1.47 mL/s, compared with 10.01 ± 0.85 mL/s in the control group, representing a difference of 4.37 mL/s. Clinically, an increase in Qmax from 10 mL/s to above 15 mL/s signifies a substantial improvement in the sensation of voiding difficulty and a qualitative leap in bladder emptying efficiency. A randomized controlled study reported that BFES reduced post-void residual volume and increased maximum urinary flow rate and voiding efficiency in patients with non-neurogenic DUA (8). In our study, the proportion of patients achieving Qmax ≥15 mL/s at 3 months postoperatively was nearly twice as high in the intervention group (39.2%) as in the control group (20.8%), directly reflecting the advantage of BFES in enhancing the “success rate” of surgery.

IPSS and QOL scores are key subjective measures for assessing outcomes in BPH patients. In this study, the IPSS in the intervention group decreased from 25.3 to 7.3, a reduction of 18 points, whereas the control group decreased from 25.0 to 10.6, a reduction of 14.4 points. Although both groups achieved significant symptomatic improvement postoperatively, the degree of improvement was substantially greater in the intervention group. A meta-analysis by Creta et al. (10) indicated that HoLEP results in greater IPSS improvement compared with medical therapy; however, the additional benefit observed in our intervention group suggests that patients can progress from “moderate symptoms” to “mild symptoms” or even an “asymptomatic” state, which translates into a meaningful impact on daily life.

The difference in QOL scores was even more striking: the intervention group's QOL score improved from 4.5 (“distressed”) preoperatively to 1.6 (“satisfied”) postoperatively, whereas the control group improved only to 2.5 (“mostly satisfied”). The transition from “distressed” to “satisfied” indicates that BFES not only improves voiding parameters but also alleviates the anxiety associated with persistent symptoms. A randomized controlled trial by Soto González et al. (11) in patients undergoing radical prostatectomy showed that early physical therapy, including BFES, facilitated urinary continence recovery and improved continence rates at 3 months postoperatively. These findings highlight that relying solely on surgery as a “one-time fix” may be insufficient for postoperative voiding recovery; active rehabilitation is equally essential. A randomized controlled trial by Lv et al. (12) in patients after pelvic floor reconstructive surgery demonstrated that BFES significantly improved voiding function, pelvic floor muscle strength, and patient-reported quality of life.

In our study, the incidences of postoperative urinary tract infection (13.7% vs. 28.6%), urinary incontinence (9.8% vs. 24.5%), and indwelling catheter reinsertion (2.0% vs. 12.2%) were significantly lower in the intervention group than in the control group. The reduction in these complications is likely attributable to improved bladder emptying efficiency conferred by BFES. Postoperative residual urine volume is a well-established risk factor for urinary tract infection (13); in our study, the intervention group had a significantly lower PVR (21.8 ± 5.8 mL) compared with the control group (40.2 ± 7.5 mL), implying a smaller “dead space” within the bladder that is less conducive to bacterial colonization. Plata et al. (14) reported that BPH patients with DUA have a higher incidence of postoperative bladder emptying difficulties. The improvement in voiding efficiency also reduced the need for catheter reinsertion. The observed reduction in urinary incontinence may be related to the enhancement of urethral sphincter and pelvic floor muscle control through BFES. A systematic review by Wu et al. (15) concluded that BFES effectively improves symptoms of urinary incontinence in women; the present study extends these findings to the male post-BPH surgery population, achieving similarly favorable outcomes. No significant differences were observed in bleeding or urethral stricture rates between the two groups, indicating that postoperative BFES does not increase the risk of perioperative bleeding or long-term urethral stricture. As a non-invasive therapy delivered via surface electrodes, BFES does not involve manipulation of the urethra or bladder, and therefore would not be expected to increase the risk of urethral injury or bleeding. This safety profile is crucial for clinical adoption, as it alleviates concerns for both patients and clinicians regarding potential risks associated with additional therapy.

From a clinical practice perspective, the BFES regimen used in this study (initiated postoperatively, three times weekly for a total of 10 sessions of 30 min each) spans approximately 3–4 weeks, coinciding with the postoperative recovery period, thus facilitating patient adherence and clinical implementation. For patients with preoperatively confirmed DUA, proactive initiation of BFES postoperatively may improve surgical outcomes, shorten the recovery process, and reduce the risk of re-hospitalization. Moreover, as a physical therapy, BFES carries no risk of drug interactions or wound infection, making it particularly suitable for elderly patients. Our findings further suggest that for patients with BPH and DUA, relying solely on surgical “obstruction relief” may be insufficient; postoperative functional rehabilitation is equally important. This concept aligns with the principles of enhanced recovery after surgery (ERAS), shifting the focus from “surgical success” to “functional recovery” and placing greater emphasis on overall patient prognosis and quality of life.

Although the present study yielded positive results, several limitations should be acknowledged. First, the retrospective design inherently carries a risk of selection bias, and despite balanced baseline characteristics between the two groups, unmeasured confounding factors cannot be entirely excluded. Second, the sample size was relatively small and derived from a single institution, limiting the generalizability of the findings. Third, the follow-up period was limited to 3 months, leaving the long-term efficacy of BFES (e.g., beyond 1 year postoperatively) uncertain. Fourth, we did not perform a subgroup analysis based on the severity of DUA, and the response to BFES may differ across different DUA subtypes. Fifth, the study did not include a group receiving BFES alone without surgery, precluding an assessment of the standalone efficacy of BFES in this population. Additionally, patient-reported outcome measures such as treatment adherence and satisfaction were not evaluated; these will be addressed in future studies.

## Conclusion

Biofeedback electrical stimulation combined with HoLEP significantly improves voiding function, clinical symptoms, and quality of life in patients with BPH and DUA, enhances bladder contractility, and reduces the risk of postoperative complications without increasing the incidence of perioperative adverse events. This combined treatment strategy offers a promising new option for the clinical management of this patient population and warrants broader implementation in clinical practice.

## Data Availability

The original contributions presented in the study are included in the article/Supplementary Material, further inquiries can be directed to the corresponding author.
